# Designing a Scale to Assess Dialectical Thinking: Link to ECERS-R Items

**DOI:** 10.3390/bs9120157

**Published:** 2019-12-14

**Authors:** Nikolay Veraksa, Olga Shiyan, Ekaterina Sviridova

**Affiliations:** 1 Faculty of Psychology, Lomonosov Moscow State University, Moscow City University, 125009 Moscow, Russia; neveraksa@gmail.com; 2 Laboratory of Child Development, Moscow City University, 129226 Moscow, Russia; olgablues@gmail.com; 3 State-Run School # 152, 125319 Moscow, Russia

**Keywords:** dialectical thinking, preschool age, assessing the quality of preschool education, the ECERS-R scale

## Abstract

Dialectical thinking is gaining wide circulation as part of personal and social preschool child development in modern society, which makes all the more urgent the task of designing a tool to evaluate the extent to which the educational environment in a pre-school establishment supports the development of dialectical thinking in preschoolers. To implement this task, the researchers analyzed the ECERS-R as a means for assessing the quality of preschool education and concluded that this tool fails to focus on rating the environment in terms of the development of dialectical thinking. N.Ye. Veraksa and E.V. Sviridova designed a tool for assessing how well the educational environment supports dialectical thinking in preschoolers (the scale of dialectical thinking support (DTS scale). The research into the use of the DTS scale was conducted in 18 preparatory groups of three educational complexes in Moscow in February–April 2019. The comparison of the results on the DTS scale and those on the ECERS-R scale made it possible to conclude that the ECERS-R scale does not differentiate between the stimulation of dialectical thinking and formally logical thinking in preschool age children. The use of the newly designed tool was justified statistically. It is noted that the teacher activity in line with the ECERS-R scale scores of “Stimulating Communication with Children”, “Books and Illustrations”, “Using Speech to Develop Cognitive Skills” may be associated with decreased levels of support for children’s dialectical thinking in preschool educational institutions. In addition, a positive relationship was found to exist between the ECERS-R score of “Care-Giver and Children Interaction” and DTS scale. The results obtained make it possible to hypothesize that there are interrelations between the development of dialectic thinking in children, on the one hand, and voluntariness and the emotional sphere, on the other.

## 1. Introduction

The problematics of dialectical thinking in preschool age children have been established over the past two centuries. For example, L.S.Vygotsky [1] was the first to systematically apply the dialectical approach to the study of various aspects of child development. He succeeded in showing the dialectical nature of child thinking [2,3,4,5,6].

J. Piaget [7,8,9,10,11] was one of the first authors to experimentally explore dialectical thinking in children. M. Basseches [12], a well known expert on adult dialectic thinking, provided the following description of J. Piaget’s scientific work: “Piaget’s general approach to epistemology was absolutely dialectical”. In his examination of human knowledge about numbers, space, causality, reality, time and so forth, he proceeded from the assumption that such knowledge can be transformed only through a dialectic process, in which developmental transformations of knowledge emerge as a result of constructive and interactive relations between people in possession of this knowledge and the world around them. This assumption allowed Piaget to believe that the dialectical analysis of the ontogenesis of knowledge (cognitive development) in children can reflect the phylogenetic roots of the knowledge that exists in adults.

Although J. Piaget did investigate elementary dialectic structures [13], he proceeded largely from the assumption that preschool children have limited intelligence. Considering children’s explanations of causality, and, in particular, their explanations of causality in conflicting situations, he came to the conclusion that preschoolers do not distinguish between artificial (human-made) and natural objects [9] and are insensitive to contradictions. Opportunities for the development of dialectical thinking were presented by A.V. Zaporozhets and G.D. Lukov [14]: they experimentally proved that preschoolers are capable of reflecting contradictory situations and understanding the causality of elementary events.

Later on, K. Riegel [15] characterized dialectical thinking as an ability to analyze an object from opposite points of view. He described dialectical operations that he believed to exist at every stage of a subject’s development. At the same time, Riegel began to consider dialectical thinking as a post-formal stage in the development of intelligence in adult subjects [16]. In connection with Rigel’s works, dialectical thinking began to be understood as a form of adult cognitive activity, which was largely to determine the research area on this issue [17,18,19,20,21,22,23,24,25].

Schoolchildren’s dialectical thinking which operates by means of dialectical logic, was considered by V.V. Davydov [26,27,28]. He proceeded from the understanding of dialectical thinking as theoretical, whose method was to ascend from the abstract to the concrete. Davydov believed that through dialectic thinking, an individual discovers in an object its concrete nature as a unity of various definitions, which reason recognizes solely in their separate aspect. However, his works failed to reveal dialectical cognitive actions and operations, which, in fact, allow the ascent from the abstract to the concrete.

In our works, we consider dialectical thinking as a method which operates by using relations of opposites [29,30,31]. We describe dialectic cognitive actions that characterize strategies for transforming conflicting problem situations, and means of dialectical thinking. As a means of dialectical thinking, we consider complex and cyclical representations. “In complex representations, an object is reflected in the totality of its various properties. Cyclic representations reflect successive changes in objects and phenomena which are characterized by marked initial and final states. Complex and cyclical representations allow preschoolers to reflect the relations of opposites” [30]. Thus, cyclical representations are considered as a structural basis for dialectical thinking in preschool children [29,32].

Since studies have shown that the development of dialectical thinking lies in the zone of the immediate development of preschool children, it is important that kindergarten teachers create the conditions for its development. In particular, this can be expressed in the fact that teachers teach children to distinguish opposite relationships, resolve conflicting situations, etc.

Given the growing interest in the issues of dialectics, this adds more urgency to the task of developing a tool to evaluate the extent to which the educational environment of a pre-school organization supports the development of dialectical thinking in preschoolers.

## 2. Materials and Methods

### 2.1. Measures

The ECERS-R Scale [33] is one of the internationally recognized tools that assess the quality of pre-school education. The scale has been tested in Russia and has proven to be a reliable and valid instrument [34]. At present, ECERS-R is subdivided into seven subscales: “Subject-Spatial Environment”; “Child Supervision and Care”; “Speech and Thinking”; “Types of Activity”; “Interaction”; “Program Structuring”; “Parent and Staff”.

The ECERS-R subscales are not directly aimed at assessing the environment in terms of possibilities for developing dialectical thinking.

With this in mind, we designed a tool for assessing the way the educational environment supports dialectic thinking in preschoolers (the scale of dialectical thinking support (DTS)). The following main indicators were selected:

1. Use and discussion of possible opposites to existing utterances and objects in the classroom and outside it (PO);
2. Search for and discuss objects and opinions that contain a contradiction in and outside the classroom (SO);
3. Discussion and consideration of the processes of change, transformation, development of surrounding objects and situations inside and outside the classroom (PoC);
4. Discussion and consideration of cyclical events inside and outside the classroom (a cycle of the seasons, times of the day, etc.) (CE);
5. The possibility of changing the object environment (COE).

Including the noted parameters in the criteria of the tool for assessing the support of dialectical thinking, we assume that support for dialectical thinking (the use and discussion of opposing factors throughout the day) can be extremely rare.

These positions of analysis and observation were constructed in accordance with the basic operations and means of dialectical thinking that has been investigated [30].

The rating system was made similar to that of the original ECERS-R scale. All this made it possible to correlate the tool we had created with the ECERS-R scale.

Observation procedure: the expert was present in the group for 4–5 h, assessing the subject-spatial environment and observing the interaction between children and adults.

### 2.2. Participants

The study was conducted in the period beginning in February 2019 and ending in April 2019 in three educational establishments in Moscow. The study involved 18 preparatory groups of state preschoolers (382 children 6–7 y.o.; M = 6.65 y.o.; 52% girls). Selected districts in Moscow were characterized by the same level of infrastructure and are designed to accommodate primarily medium-income families. The districts were selected using postal codes.

Parents or caregivers provided written informed consent for their child to take part. The study was approved by the Ethics Committee of the Russian Psychological Society.

The study was conducted in the form of observation.

## 3. Results and Discussion

In general, groups received low scores on the scale (maximum score is 7) (see Table 1.).

Based on these data, it is possible to say that the pre-school educational environment does not provide sufficient stimulation to preschool children. The observations showed a very low presence of conditions that corresponded to the two main indicators of the scale: “Use of, and discussion about, possible opposites in existing utterances and objects in and outside the classroom” and “Searching for and discussing objects and opinions containing a contradiction in and outside the classroom”. In particular, it is important to note that problem situations that arise in children’s daily life are not used as material for discussion and reasoning. Conversely, teachers tend to either ignore the arising contradictions or criticize children for the very fixation of uncertain situations (when an object turns out to be good and bad at the same time), demanding that children’s opinion be unambiguous. Despite the fact that various kinds of transformations, including cyclical ones that occur in nature or with objects around the children or with the children themselves, are often fixed by the teacher, the latter consider them as a sequence of separate states rather than moments of development. It is also important to note that in most cases, the group’s object-spatial environment, per se, stays put and does not change to suit children’s interests, which means children are less likely to experience change and development, as well as their own impact on change and subjective action.

Based on these outcomes, it is possible to talk about teachers’ non-reflective use of reasoning.

In the present study, our task was to analyze the diagnostic capabilities of the scale of support for children’s dialectical thinking in the preschool educational environment. For this purpose, we compared the results obtained using the ECERS-R scale with those using the DTS scale.

For statistical data processing, we used the two-sided Spearman rank correlation coefficient. There were several considerations in making this choice. First, the selected indicator does not require that the data correspond to any distribution type, which is convenient in the case of a small sample (18 groups under survey). Secondly, this indicator analyzes both data presented in the ordinal scale and our results. Thirdly, the two-sided coefficient was chosen based on the relatively limited values of the indicators for the compared scales.

The data of the primary analysis are displayed in Table 2.

As can be seen from Table 2, it presents one significant correlation coefficient—525 (significance at the level of 0.05)—between Subscale 3 “Speech and Thinking” and the DTS scale. The coefficient is negative. For a more detailed analysis of the results, consider the Spearman correlation coefficient values between the data obtained using the DTS scale and those using the Subscale “Speech and Thinking”. The results are presented in Table 3.

Note that the module’s highest coefficient values are obtained by comparing them with those of “Stimulation of Communication with Children”, “Books and Illustrations”, “Use of Speech for Developing Cognitive Skills”. Although these data are not significant, it can be assumed that they reflect a trend whereby an increase in the score for these indicators is accompanied by a decrease in the score on the DTS scale. The trend itself can be interpreted with a grain of salt as one pertaining to teachers who ignore the contradictory nature of objects and situations when stimulating communication between children, both during play and group classes. This spells out a practice that has taken root in the preschool education system to organize interaction in a way that rules out search for opposing trends. Most likely, this indicator testifies to teachers’ desire to smooth out contradictions in communication between children instead of emphasizing them. Therefore, a teacher’s work to stimulate communication in children may be interpreted as positive by experts when assessing it according to the proposed ECERS-R scale parameters but simultaneously, this reduces the possibilities of spontaneous support for dialectical thinking.

With regard to a possible trend for a negative relationship according to other subscale indicators, we can assume that there exists a similar trend. As far as “Books and illustrations” goes, a high point is scored in case a large variety of literature is used and these books are available to children. It is quite possible that the literature, books, illustrations, etc., available to the groups may not substantively support their search for opposites and their consideration of contradictions, processes of development, modification, transformation and so forth; in other words, they do not stimulate all the processes that we associate with the possibilities of developing dialectical thinking. The indicator “Use of Speech for Developing Cognitive Skills” focuses on finding out the extent to which a teacher stimulates the speaking of logical connections, children’s thoughts, including their own experience. The presence of high scores according to this indicator may stem from the support of formal logical reasoning and lead to a decrease in, albeit spontaneous, cases of stimulation of dialectical reflections.

Thus, the preliminary results obtained allow us to formulate a hypothesis that the ECERS-R scale does not differentiate between support for dialectical and formal-logical thinking.

We observed a pattern for establishing a relationship between the outcomes obtained using the DTS scale and the “Interaction” ECERS-R subscale. The values of the Spearman rank correlation coefficient are presented in Table 4.

An important outcome is the presence of a significant relationship between the ECERS-R indicator “Staff and Child Interaction” and the DTS scale. When rated highly, this indicator describes the teacher’s positive informal attitude towards children; his emotional involvement in the process of communicating with the children. It includes indicators such as “Educators sympathize with and help those children who are upset, hurt or angry” and “Educators encourage mutual respect between children and adults, for example, when answering questions, they do not begin to speak until the child has completed his utterance, etc.” This kind of talk inevitably concerns the resolution of emerging problem and conflict situations, as well as situations of uncertainty. It can be assumed with a high degree of probability that this way of interaction with children and this level of involvement in their professional activities allow teachers to perform, albeit spontaneously, actions that support the development of dialectical thinking. In groups that received high scores for this indicator, the teachers did not limit their interaction with children only to the requirements of the educational program in and outside the classroom; they brought their personal experience to interaction with children.

Another interesting trend is that reflected in the insignificant correlation between the ECERS-R indicator “discipline” and the data on the DTS scale. The “discipline” indicator implies children’s active involvement in solving conflict situations, which can, by itself, stimulate the development of dialectical thinking. A similar assumption can be formulated based on the fact that there is a very insignificant relationship between the ECERS-R indicator “The Interaction between Children” and the DTS scale. The high results for this indicator also suggest teachers’ assistance in overcoming children’s conflicts and finding ways to build cooperation.

## 4. Conclusions

Based on this study, we can draw the following conclusions. First of all, it is necessary to point out the validity of using the tool we developed to assess the support of the preschool educational environment for dialectic thinking. The use of this tool has shown that the ECERS-R scale does not distinguish between the stimulation of dialectical thinking and that of formally logical thinking in preschoolers.

In line with the ECERS-R scale indicators of “Stimulation of Communication with Children”, “Books and illustrations”, “Use of Speech for Developing Cognitive Skills”, teachers’ activity may be associated with a decrease in the levels of support for dialectical thinking in children at preschool educational institutions.

Another important fact is the establishment of a positive relationship between the ECERS-R scale indicator “Staff and Child Interaction” and the scale of support for dialectical thinking.

The results obtained make it possible to hypothesize that there are interrelations between the development of dialectic thinking in children, on the one hand, and voluntariness and the emotional sphere, on the other.

## Figures and Tables

**Table 1:** Distribution of dialectical thinking support (DTS) scores among groups.

General Score, DTS Scale	Number of Groups
4	1
3	3
2	2
1	12

**Table 2:** Spearman’s correlation coefficient when comparing the total points scored using the developed tool with separate ECERS-R subscales.

	1	2	3	4	5	6	7
Final score according to the developed tool (“Stimulation of dialectical thinking”)	−0.116	−0.116	−0.525 *	0.127	0.460	0.250	0.100
The average value of points in the subscales: 1 = “Subject-spatial environment”; 2 = “Child supervision and childcare”; 3 = “Speech and thinking”; 4 = “Types of Activity”; 5 = “Interaction”; 6 = “Program Structuring”; 7 = “Parents and staff.”							

* The correlation is significant at 0.05 (two-way).

**Table 3:** Spearman’s correlation coefficient when comparing the total scores for the tool developed with the ECERS-R subscale indicators ‘Speech and Thinking’.

	1	2	3	4
The final score according to the developed tool (“Stimulation of Dialectical Thinking”)	−0.321	−0.443	−0.307	0.163
Scores according the indicators of the “Speech and Thinking” subscale: 1 = “Books and Illustrations”; 2 = “Stimulating Communication between Children”; 3 = “Using Speech to Develop Cognitive Skills”; 4 = “Daily Use of Speech.”				

**Table 4:** Spearman’s correlation coefficient when comparing the total scores for the designed tool with the indicators of the ‘Interaction’ ECERS-R subscale.

	1	2	3	4
1 The final score according to the designed tool (“Stimulation of dialectical thinking”)	−0.321	−0.44 *	−0.307	0.163
Scores in terms of the “Interaction” subscale: 1 = “General Supervision over Large Motor Skills Development in Children”; 2 = “General Child Care”; 3 = “Discipline”; 4 = “Staff and Child Interaction”; 5 = “Interaction between Children”.				

* The correlation is significant at 0.05 (two-way).
